# An integrative framework to identify cell death-related microRNAs in esophageal squamous cell carcinoma

**DOI:** 10.18632/oncotarget.10779

**Published:** 2016-07-22

**Authors:** Bing-Li Wu, Dong Wang, Wen-Jing Bai, Fan Zhang, Xing Zhao, Ying Yi, Ting Zhang, Wen-Jun Shen, En-Min Li, Li-Yan Xu, Jian-Zhen Xu

**Affiliations:** ^1^ The Key Laboratory of Molecular Biology for High Cancer Incidence Coastal Chaoshan Area, Shantou University Medical College, Shantou 515041, China; ^2^ Computational Systems Biology Laboratory, Department of Biochemistry and Molecular Biology, University of Georgia, Athens, GA 30602, USA; ^3^ Department of Biochemistry and Molecular Biology, Shantou University Medical College, Shantou 515041, China; ^4^ Institute of Oncologic Pathology, Shantou University Medical College, Shantou 515041, China; ^5^ Department of Bioinformatics, Shantou University Medical College, Shantou 515041, China; ^6^ College of Bioinformatics Science and Technology, Harbin Medical University, Harbin, Heilongjiang 150086, China

**Keywords:** autophagy, apoptosis, cell death, miRNA, esophageal squamous cell carcinoma

## Abstract

Cell death is a critical biological process involved in many important functions, and defects in this system are usually linked with numerous human diseases including cancers. Esophageal squamous cell carcinoma is one of the most chemo- and biological therapy resistant cancers. Based on knowledge repository and four miRNAs profiling data, we proposed a general framework to hunt for cell death miRNAs in a context dependent manner. We predicted 12 candidate miRNAs from hundreds of others. Follow-up experimental verification of 7 miRNAs indicated at least 3 miRNAs (MIR20b, MIR498 and MIR196) were involved in both apoptosis and autophagy processes. These results indicated miRNAs intimately connected the two cell death modules in esophageal squamous cell carcinoma. This integrative framework can also be easily extended to identify miRNAs in other key cellular signaling pathways or may find conditional specific miRNAs in other cancer types.

## INTRODUCTION

MicroRNAs (miRNAs) are a group of ~21 nucleotide long endogenous noncoding RNAs, which play an important role in a broad range of biological processes [1–6]. During the past several years, great interests in defining the Cell Death related MiRNAs (CD-MiRs) in human health and disease have become widespread [7–9]. A variety of experimentally confirmed CD-miRs have been documented into our recently established ncRDeathDB resource (www.rna-society.org/ncrdeathdb) [10]. Despite great success, there still remain two research obstacles. Firstly, the traditional approaches, which are usually experimentally screened for many candidate miRNAs, are labor-intensive and time-consuming. Secondly and most importantly, the same miRNA is commonly seen to have different or even opposing effects under diverse cell conditions. For example, while MIR95 is highly expressed in both prostate and lung cancers and have oncogenic function [11, 12]; the anti-proliferative role of MIR95 was observed in breast cancer MCF-7 cells [13]. These observations indicated that context and/or cell types dependent CD-MiRs are of great value and should be the ultimate research goal. In genomic era, large sequencing projects have produced plenty of omics datasets including expression profiling and gene annotation resources. These omics data provide us a good opportunity to conduct an alternative ‘data-driven’ strategy to *in silico* hunt for CD-MiRs on a large scale. Therefore, we have proposed an integrative framework to identify CD-MiRs in a context dependent manner. We reasoned that a functional miRNAs should have tight control over the backbone of cell death modules such as apoptosis or autophagy pathways via its targeting genes, *i.e*. it should have significantly higher number of gene targets within the focused cell death pathway. This genome knowledge filtering process can immediately eliminate hundreds of irrelevant miRNAs. The resulting candidate CD-MiRs should be functional only if it is activated or repressed, which can be monitored by expression profiling platform. Thus combining the differentially expressed miRNAs with these functional related candidate CD-MiRs, which are filtered by knowledge resources, we can predict a handful of context specific CD-MiRs, that can be further subjected to follow-up experimental confirmation.

Esophageal cancer is a serious malignancy with respect to prognosis and mortality rate. It is among the five leading cancer types for the cancer deaths in males of mid-age in US, the 5-year relative survival rates is less than 20%[14]. In China, esophageal cancer is the fourth most frequently diagnosed cancer and the fourth leading cause of cancer death [15]. In the last two years, several functional miRNAs have been identified in esophageal squamous cell carcinoma (ESCC) [16–18], but the role of miRNAs in governing cell death routes remains elusive. In order to accelerate the progress, we have tested our computational frameworks to find CD-MiRs in ESCC.

## RESULTS

### A computational framework to hunt for human cell death related miRNAs in esophageal cancers

**Figure 1:**
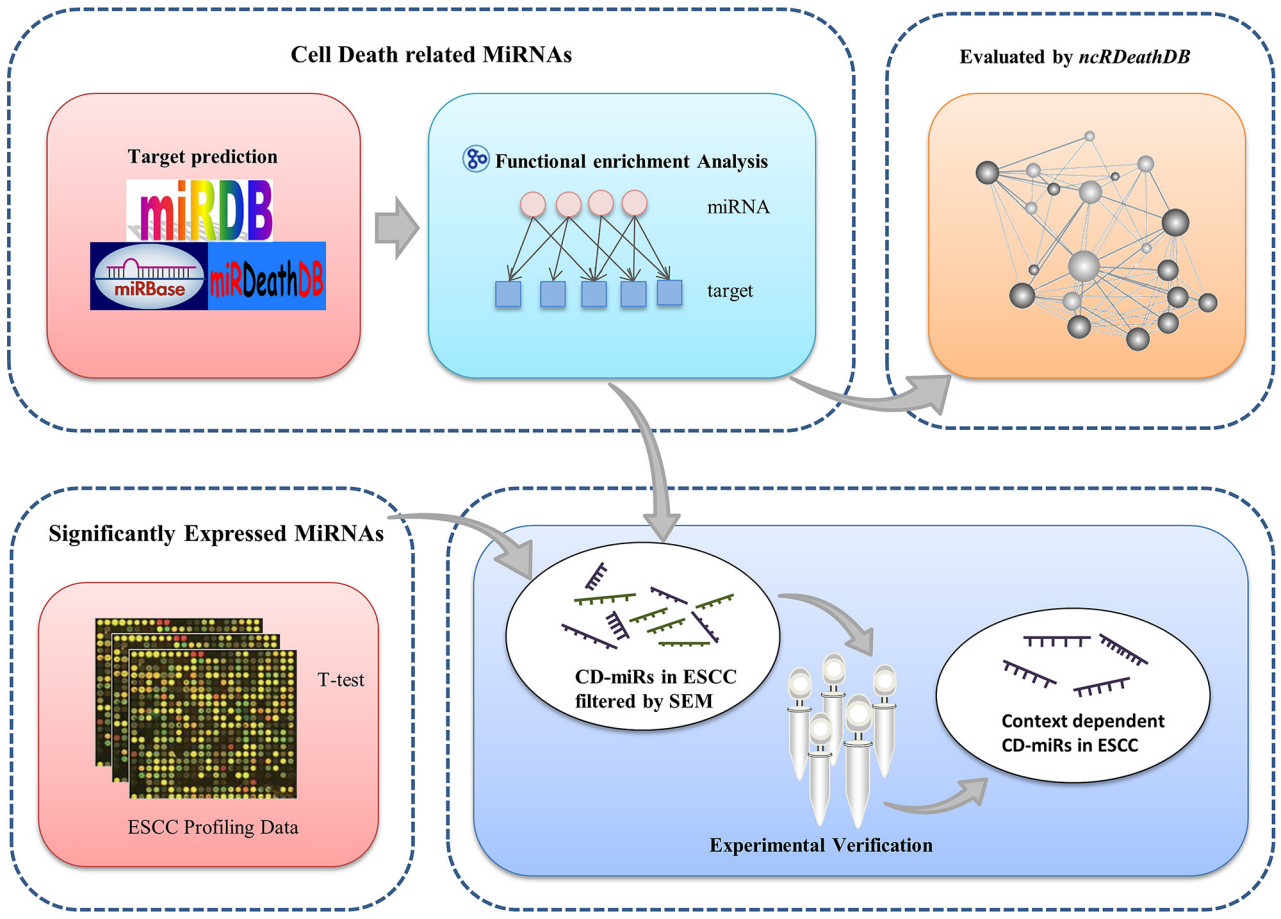
Overview of analytical scheme for cell death related miRNAs prediction

To hunt for human cell death miRNAs, we initially extracted the annotated human miRNAs from the miRBase database. As indicated in Figure 1, for each of the miRNAs, we found its predicted target genes according to the intersection genes set of miRDB and miRanda algorithms. We reasoned that if one miRNA has multiple targeting genes within the same cell death related pathway, it should has more chance to regulate that death process. Based on this concept, we searched for the miRNAs whose targeting genes are significantly enriched in GO terms “apoptotic process” (GO:0006915) and “autophagy” (GO:0006914) or both of them. We defined these set of miRNAs as cell death related miRNAs (CD-miRs). At the other hand, we selected differentially expressed miRNAs based on 3 pair-matched microarray datasets and one paired esophageal cancer RNA-seq dataset.

In order to hunt for esophageal cancers specifically expressed CD-miRs, we then combined this set of differentially expressed miRNAs with cell death related miRNAs to obtain a list of esophageal cancers specifically expressed cell death miRNAs. Our prediction results were further evaluated by different biological experiments.

### Prediction and evaluation of predicted miRNAs based on known human cell death miRNAs

Based on our GO enrichment analysis, 515 miRNAs with targets gene enriched in functional terms ‘apoptosis’, ‘autophagy’ or both of them, were identified as cell death related miRNAs (Supplementary File 2). From our manually collection of ncRNADB database, we compiled a list of 221 known human cell death miRNAs. Combining this list of miRNAs with the initial 629 miRNAs resulted in 176 miRNAs. It includes 137 apoptosis related miRNAs, 6 autophagy related miRNAs and 33 dual functional miRNAs (M176) (Supplementary File 3). All of these 176 miRNAs have been confirmed in at least one experimental condition and regarded as “golden criterion”. Based on this data, we set out to evaluate the efficiency of our prediction. Of the M176 known miRNAs, we successfully identified 158 true cell death miRNAs, but at the cost of another 352 additional predictions. To assess the statistical significance, 176 miRNAs was random sampled from the initial 629 miRNAs for 10,000 times, and the fraction of 10,000 random sampling having equal to or larger than 158 was reported as *P* value. Compared with the random sampling data, our prediction results are significantly enriched with cell death related miRNAs (*p*<3.8E-4). To note that, since the known cell death miRNAs is limited and currently only a small set of them have been identified, so a false positive in our prediction result may be demonstrated as a *bona fide* one as time goes.

### Combined with miRNAs profiling data to identify esophageal cancers specific CD-miRs

In order to identify esophageal cancers specific CD-miRs, we first identified the Differentially Expressed MiRNAs (DEMs) in three microarray based and one RNA-seq based ESCC profiling data. Table 1 shows the numbers of DEMs, the numbers of both differentially expressed and cell death related miRNAs, and novel candidates identified in four esophageal cancers profiling datasets. As seen from it, there are 30,23,25 and 47 differentially expressed miRNAs were found respectively in these profiling datasets. Among them there are 27,20,21 and 40 were overlapped with our M176 known cell death related miRNAs. Except for the known cell death related miRNAs, there remains 12 novel miRNAs which are both differentially expressed and are cell death related. These miRNAs are then subjected to literature survey and experimental verification.

**Table 1:** Number of differentially expressed miRNAs, both DEMs and CD-miRs and novel candidates

Datasets	Differentially Expressed miRNAs	Both DEM and CD-miRs	Novel	miRNA symbols
Batch272	30	27	5	HSA-MIR363
				HSA-MIR337
				HSA-MIR30E
				HSA-MIR216A
				HSA-MIR20B
GSE6188	23	20	1	HSA-MIR422A
GSE13937	25	21	4	HSA-MIR202
				HSA-MIR196A
				HSA-MIR410
				HSA-MIR498
GSE43732	47	40	2	HSA-MIR140
				HSA-MIR28

### Experimental verification of CD-miRs

**Figure 2:**
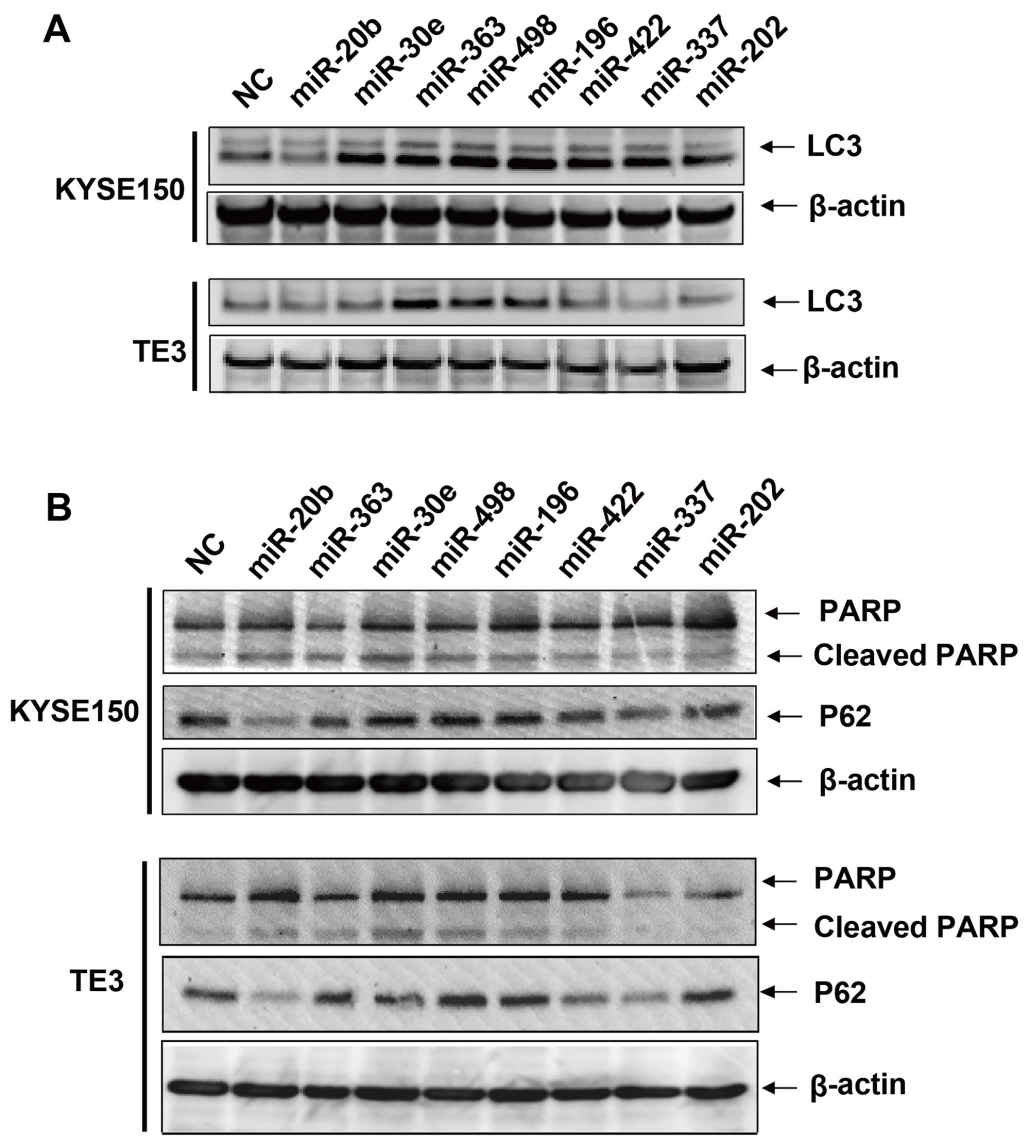
Immunoblots of LC3, P62, PARP and cleaved PARP protein after transfection of predicted cell death related miRNAs KYSE150 or TE3 cells were transfected with 30 μM of predicted cell death related miRNAs or NC. After 48 hrs, cell pellets were collected and total proteins were extracted. **A.** Immunoblots of LC3 and β-actin loading controls. **B.** Immunoblots of P62, PARP, cleaved PARP and β-actin loading controls.

Literature survey of recent published reports indicated 5 miRNAs among the 12 predicted CD-miRs, namely MIR216a/MIR140/MIR28/MIR363/MIR410, have been associated with apoptosis or autophagy in various experimental settings [17, 21–24]. So use of MIR363 as a positive control, we set up experimental assays to confirm whether the other 7 predicted miRNAs had an effect on cell death related processes. Esophageal cancers cells were respectively transfected with synthetized miRNA and the negative control. The autophagy marker LC3 and p62 were firstly assessed by western blotting. In esophageal cancers cell line KYSE150, as shown in the up panel of Figure 2A, over-expression of MIR30e, MIR363, MIR498, MIR196, MIR422, MIR337 or MIR202 remarkably increase the LC3-I to LC3-II conversion whereas miR-20b modestly decrease. In addition to LC3, the sequestosome 1(SQSTM1/P62) protein were accumulated when MIR30e, MIR363, MIR498 or MIR196 were transfected whereas MIR20b significantly decreased (up panel of Figure 2B). In another esophageal cancers cell line TE3, transfection of MIR20b, MIR363, MIR498 or MIR196 affected the autophagy when monitoring both LC3 and p62 (down panel of Figure 2A and Figure 2B).

**Figure 3:**
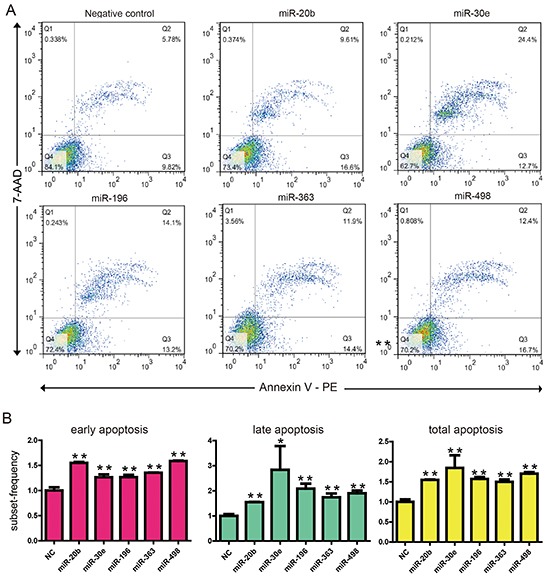
KYSE150 cells were transfected with 30 nM of 5 cell death related miRNAs or NC After 48 hr, cells were subjected to flow cytometric analysis with the use of FITC Annexin V Apoptosis Detection Kit. **A.** Representative graphs are shown. **B.** The flow cytometric analysis experiments had been repeated three times and quantified. The amounts of apoptosis induced by negative oligonucleotide control and cell death related miRNAs are shown. Data are expressed as mean±S.E.M. **p < 0.01. (c). *p < 0.05.

We also investigated the effect of these miRNAs on apoptosis via apoptotic marker poly (ADP-ribose) polymerase 1 (PARP). The cleaved PARP were modestly but significantly increased when transfection of MIR20b, MIR30e, MIR498 or MIR196 in both cell lines (Figure 2B). To confirm this result, we used a flow cytometer assay to check if the overexpression of these four cell death related miRNAs induced apoptosis. As can be seen from Figure 3, overexpression of these cell death related miRNAs resulted in a modestly but significantly increase in the early apoptotic fraction. The most efficient miRNAs is MIR498 since transfection of it increased the early apoptotic fraction from 9.82% to 16.7% compared to negative control (Figure 3). In addition, miRNA transfection also leads to various degree of increase of cell fraction in late apoptosis or already dead status (Figure 3). Collectively, above results suggested that the four predicted miRNAs, MIR20b, MIR30e, MIR498 and MIR196 may be involved in the apoptotic pathway in esophageal cancers cells.

## DISCUSSION

Resisting cell death remains as one of the hallmarks of cancers [25]. At one hand, dysfunction in apoptotic machinery hampered damaged cells into apoptotic suicides; at the other hand, cytotoxic stress, for example chemotherapy, will elevate level of autophagy to protect cells from environmental stress or even promote the cancer cell proliferation. After decades of studies, the genes functioning in cell death modules, such as apoptosis and autophagy, have been deciphered into great details [26, 27]. These cell death related genes can be classified as upstream regulators and downstream effectors components. They interact with each other to sense and respond to the intracellular and extracellular signals, sustaining cellular homeostasis

Esophageal cancer includes two major histologic types: esophageal squamous cell carcinoma (ESCC) and esophageal adenocarcinoma (EAC). The histological type of esophageal cancer is geographic and race dependent, *i.e*. esophageal squamous cell carcinoma (ESCC) is the predominant histologic type in Asian, while the EAC has become the leading cause of esophageal cancer in the United States [28, 29]. The molecular mechanisms of ESCC development is not fully understood currently, but emerging studies pointed out that cell death related mechanism may actively associate within the pathogenesis. For example, recent large exon sequencing project revealed that genes involved in cell cycle and apoptosis regulation were mutated in 99% of cases by somatic alterations [30, 31]. In addition to genetic defects, alterations in genes in cell death pathways also contribute to the disease. For example, pharmacological and genetic inhibition of autophagy enhanced the resveratrol-induced cytotoxicity to the ESCC cells [32]. A recent study also indicated that AZD2281, Novel poly (ADP-ribose) polymerase inhibitor enhances radio-sensitivity in esophageal squamous cancer cells [32, 33]. In this project, we proposed an integrative framework to identify the CD-MiRs in ESCC. It efficiently makes use of the public omics database to generate a handful of candidate CD-MiRs, which can be experimentally tested further. We revealed a network of 515 CD-MiRs via miRNAs-targets and knowledge database based refinements. These candidate miRNAs may function under certain conditions. Combined with ESCC profiling data, we narrowed down to 12 novel ESCC specific miRNAs. Follow-up experiments indicated transfection of MIR20b, MIR30e, MIR498 and MIR196 affected the apoptotic pathway in esophageal cancers cells. To investigate whether or not these miRNAs affect autophagy process, we use protein markers, LC3 and P62, to monitor the autophagy changes after transfection of predicted cell death related miRNAs. The results indicated at least 3 miRNAs (MIR20b, MIR498 and MIR196) were involved in cell death in two esophageal cancers cell lines. This high concordance suggested the good performance of our framework.

Interestingly, three out of the four apoptotic related miRNAs (MIR20b, MIR498 and MIR196), can modulate both autophagy and apoptosis processes. Previously we and others have observed that, some miRNAs are involved in both autophagy and apoptosis processes [8, 34].We defined this type of miRNAs as ‘dual functional miRNAs’ in cell death process. ‘Dual functional miRNAs’ can simultaneously regulate two or more targets, while these target protein play a role in either autophagy or apoptosis respectively. For example, MIR101 can modulate MCL1 (myeloid cell leukemia sequence 1) therefore promotes apoptosis [35]. On the other hand, MIR101 can also negatively modulate the autophagy flux via inhibiting autophagy-related protein RAB5A [36]. Sometimes ‘Dual functional miRNAs’ can target common regulators of both autophagy and apoptosis [37]. This miRNA mediated crosstalk provides a novel communication mechanism between different module of cell death [8, 34]. Depending on the dominant target genes of miRNAs, they can modulate the two statuses simultaneously. Thus miRNAs functions as a molecular switch between autophagy and apoptosis in cancer cells. The next step towards detailed understanding of the 3 novel ‘dual role miRNAs’ would be to identify their potential targets in each of the death modules and to elucidate the molecular mechanism underlying the crosstalk.

It is should be pointed out that this frame work is flexible, dynamic, and easily to adapt to other experimental conditions. Although the framework was initially established for identifying cell death related miRNAs, it can be straightly extend to identify miRNAs in other key cellular signaling pathways such as cell cycle or metastasis, as long as the corresponding functional knowledge annotations are available. Furthermore, the identified cell death miRNAs can easily be integrated with cancer specifically profiling data to filter out the conditionally functioned CD-MiRs.

## MATERIALS AND METHODS

### Data source and miRNA expression data

miRNAs annotation data were download from miRBase 21 (http://www.mirbase.org/). The intersection of miRDB (www.mirdb.org) and miRanda (August 2010 Release) prediction was used as miRNAs targets prediction data. It totally includes 236,154 miRNA–target gene pairs, which involved 629 miRNAs and 16,163 target genes. In order to collect all known miRNAs involved in programmed cell death, we have established miRDeathDB [19] (http://rna-world.org/mirdeathdb/) three years ago. miRDeathDB is a knowledge database that integrates information for experimentally identified miRNAs and their targets in cell death network. Now it has evolved into the updated version of ncRDeathDB (www.rna-society.org/ncrdeathdb) [20]. As of January 2015, ncRNADB provided over one thousand human miRNAs-targets entries, which were used in this study. Genes annotated in Gene Ontology (GO) term ‘apoptotic process’ (GO:0006915) and ‘autophagy’ (GO:0006914) were used as cell death relevant genes. It could be downloaded from Amigo application (http://amigo.geneontology.org/amigo). After removing the redundant genes, there are 807 apoptotic genes, 114 autophagic related genes and 25 dual functional genes (Supplementary File 1). For miRNA expression data in esophageal cancers, we specifically selected pair-matched datasets in which the normal samples were taken from the same patients with the cancer samples. This can remove potential compound factors such as familial effects, individual effects and environmental differences. With this criterion, we have collected 3 pair-matched microarray datasets from Gene Expression Omnibus (GEO, http://www.ncbi.nlm.nih.gov/geo/). GSE13937 includes 76 US and Japanese patients enrolled in 3 distinct cohorts; GSE6188 for 104 normal and 151 disease samples; and GSE43732 for 119 normal and 119 cancer samples. We also used one paired esophageal cancer RNA-seq dataset from The Cancer Genome Atlas (TCGA, http://cancergenome.nih.gov/), including 7 normal and 30 cancer samples in Batch272. Each normalized dataset was then selected the significantly expressed miRNAs by two-tailed t-test with a simple *p*<0.01 control.

### Prediction framework

In this work, for each of the miRNAs annotated in miRBase, we first found its potential targets genes via the intersection of miRDB and miRanda prediction. These candidate genes were further filtered by GO ontology terms via hyper-geometric test. The P-value of the test statistic is calculated as follows:

$$\text{p}=1-\sum_{i=0}^{x-1}\frac{{(}_{i}^{M}){(}_{n-i}^{N-M})}{{(}_{n}^{N})}$$

Where the N is the total number of human genes contained in all GO terms, and M represents the number of cell death related human genes in GO. *n* represents the number of target genes of a certain miRNA, while *x* represents the number of cell death related human genes targeted by the miRNA. Only the miRNAs with the targets genes significantly enriched in GO terms “apoptosis” or “autophagy” or both of them were used in the follow-up studies (*p*<0.05 and *x*>1).

In order to evaluate the efficiency of our method, we have random sample equal number of miRNAs from the miRNAs list with targeting prediction. To assess the statistical significance of the enrichment analysis for miRNA, the same number of miRNAs enriched in the GO terms were random sampled 10,000 times, then the fraction of 10,000 random sampling having equal to or larger than the number of predicted CD-miRs was reported as *P* value.

Since our research aim is to find miRNAs in esophageal cancers, cell death related miRNAs were further filtered by miRNA expression profiling data. The cell death related miRNAs, which are also significantly expressed in at least one of the four sets of profiling data (*p*<0.01), are defined as esophageal cancers specific CD-miRs.

### Cell lines and cell culture

Two human ESCC cell lines were utilized in this study. KYSE150 cell line was generous gifts from Professor Dong Xie (Institute for Nutritional Sciences, Shanghai Institutes for Biological Sciences, Chinese Academy of Sciences, China), and the TE3 cell line was a gift from Professor Mingzhou Guo (Department of Gastroenterology & Hepatology, Chinese PLA General Hospital, China). KYSE150 and TE3 cells were cultured in Roswell Park Memorial Institute (RPMI) 1640 medium (HYCLONE, USA) with 10% fetal bovine serum.

### MiRNA transfection

The synthetized miRNA including the negative control were purchased from Ambion (Life Technologies, USA). 1×10^6^ KYSE150 or TE3 cells were plated in each well of 6-well plate 24 h prior to transfection. When the cells reached 60-80% confluent, the miRNAs were transfected to a final concentration of 30μM, using Lipofectamine™ RNAiMAX (Life Technologies) according to the manufacturer's recommendations.

### Western blotting

The total protein was collected after 48h by RIPA buffer containing protease inhibitor cocktail (Thermo fisher, USA). Whole cell protein extracts were boiled with SDS sample buffer and separated on 12% SDS-PAGE followed transferred onto a PVDF membrane (Roch, USA). The membranes were blocked in 5% nonfat milk for 1 h, incubated with antibodies against PARP (1:1000, CST, USA), p62 and LC3 (1:1000, CST, USA) and β-actin (1:1000, Santa Cruz Biotechnology, USA), respectively, according to the manufacturer's recommendations. The membranes were then washed and incubated with secondary antibody coupled to horseradish peroxidase for 2 h. The proteins were detected with western blot luminol reagent (Santa Cruz Biotechnology, USA) using a FluorChemTM IS-8900 (Alpha Innotech, USA).

### Flow cytometry

The apoptosis caused by the transfection of miRNAs were detected by using FITC Annexin V Apoptosis Detection Kit (BD Pharmingen™, cat#556547) according to the manufacture's protocol. Briefly, ESCC KYSE150 esophageal cancers cells were transfected with the miRNAs to a final concentration of 30 μM. The cells were collected after 48h and washed twice with cold PBS, and then re-suspended in 1X Binding Buffer to a concentration of 1 × 10^6^ cells/ml. 100 μl of the solution (1 × 10^5^ cells) were transferred o a 5 ml culture tube. 5 μl of FITC Annexin V and 5 μl PI were added and gently vortexed and incubate for 15 min at room temperature in the dark. 400 μl of 1X Binding Buffer to each tube and analyzed by a BD LSR benchtop flow cytometry within 1 hour. The experiments were repeated at least three times.

## SUPPLEMENTARY MATERIALS TABLES









## Abbreviations

ESCC: esophageal squamous cell carcinoma
GO: gene ontology
SQSTM1/P62: sequestosome 1
PARP: poly (ADP-ribose) polymerase 1
LC3: microtubule-associated protein 1 light chain 3
