# Supplementary material for: An integrative framework to identify cell death-related microRNAs in esophageal squamous cell carcinoma
Source: Oncotarget. 2016 Jul 22;7(35):56758–66. doi: 10.18632/oncotarget.10779 (PMC5302951; doi:10.18632/oncotarget.10779)
Supplement: Supplementary file 1 [file oncotarget-07-56758-s001.pdf]

An integrative framework to identify cell death-related microRNAs in esophageal squamous cell carcinoma

SUPPLEMENTARY TABLES

Supplementary File 1: List of apoptotic genes, autophagy genes and dual functional genes.

See Supplementary File 1

Supplementary File 2: List of predicted cell death related miRNAs.

See Supplementary File 2

Supplementary File 3: List of known cell death related miRNAs.

See Supplementary File 3
